# Toward Cross-Hospital Deployment of Natural Language Processing Systems: Model Development and Validation of Fine-Tuned Large Language Models for Disease Name Recognition in Japanese

**DOI:** 10.2196/76773

**Published:** 2025-07-08

**Authors:** Seiji Shimizu, Tomohiro Nishiyama, Hiroyuki Nagai, Shoko Wakamiya, Eiji Aramaki

**Affiliations:** 1 Nara Institute of Science and Technology, 8916-5, Takayama-cho, Ikoma-shi, Nara, 630-0192, Japan, 81 743-72-5250

**Keywords:** clinical NLP, Japanese language, named entity recognition, large language models, out-of-domain robustness, clinical corpus, clinical natural language processing

## Abstract

**Background:**

Disease name recognition is a fundamental task in clinical natural language processing, enabling the extraction of critical patient information from electronic health records. While recent advances in large language models (LLMs) have shown promise, most evaluations have focused on English, and little is known about their robustness in low-resource languages such as Japanese. In particular, whether these models can perform reliably on previously unseen in-hospital data, which differs from training data in writing styles and clinical contexts, has not been thoroughly investigated.

**Objective:**

This study evaluated the robustness of fine-tuned LLMs for disease name recognition in Japanese clinical notes, with a particular focus on their performance on in-hospital data that was not included during training.

**Methods:**

We used two corpora for this study: (1) a publicly available set of Japanese case reports denoted as CR, and (2) a newly constructed corpus of progress notes, denoted as PN, written by ten physicians to capture stylistic variations of in-hospital clinical notes. To reflect real-world deployment scenarios, we first fine-tuned models on CR. Specifically, we compared a LLM and a baseline-masked language model (MLM). These models were then evaluated under two conditions: (1) on CR, representing the in-domain (ID) setting with the same document type, similar to training, and (2) on PN, representing the out-of-domain (OOD) setting with a different document type. Robustness was assessed by calculating the performance gap (ie, the performance drop from in-domain to out-of-domain settings).

**Results:**

The LLM demonstrated greater robustness, with a smaller performance gap in *F*_1_-scores (ID–OOD = −8.6) compared to the MLM baseline performance (ID–OOD = −13.9). This indicated more stable performance across ID and OOD settings, highlighting the effectiveness of fine-tuned LLMs for reliable use in diverse clinical settings.

**Conclusions:**

Fine-tuned LLMs demonstrate superior robustness for disease name recognition in Japanese clinical notes, with a smaller performance gap. These findings highlight the potential of LLMs as reliable tools for clinical natural language processing in low-resource language settings and support their deployment in real-world health care applications, where diversity in documentation is inevitable.

## Introduction

Clinical notes contain a vast amount of information that is not captured in structured fields of electronic health records (EHRs) [1,2]. Natural language processing (NLP) techniques have become essential for unlocking this rich, unstructured data [3].

Among these, named entity recognition (NER)—a task that identifies clinical entities such as disease names in text—plays a vital role in extracting key clinical information, which is essential for understanding patients’ medical conditions [4-6]. For instance, disease name recognition can be leveraged to detect adverse drug reactions from EHRs for post-marketing surveillance [7].

Recent advances leveraging fine-tuned masked language models (MLMs) such as BERT, have achieved state-of-the-art performance in clinical NER tasks, often outperforming prompt-based in-context learning (ICL) of large language models (LLMs) [8,9]. However, MLMs fine-tuned for disease name recognition tend to experience notable performance drops on unseen in-hospital data [10,11]. Given that clinical NLP systems are expected to operate reliably across diverse clinical settings, understanding and improving robustness—that is, whether models can perform reliably on previously unseen clinical notes—is a critical research objective.

When fine-tuned, LLMs have shown competitive or slightly superior performance compared to MLMs in NER tasks [12]. Given their exposure to a broader and more diverse range of linguistic patterns during pretraining, LLMs are expected to exhibit resilience to stylistic variations. However, the extent to which fine-tuning improves their robustness over MLMs remains underexplored, particularly in languages other than English [13]. One reason for this research gap is the lack of corpora that reflect the realistic documentation styles of in-hospital clinical notes. These data are challenging to obtain due to privacy concerns and institutional restrictions.

In this study, we investigate the robustness of fine-tuned LLMs with a focus on disease name recognition in Japanese clinical notes. To facilitate an evaluation of robustness to unseen in-hospital data, we constructed a dedicated clinical corpus comprising progress notes (PN) authored by ten individual physicians from different clinical institutions, reflecting diverse in-hospital documentation styles. To reflect real-world deployment scenarios, we trained the models on publicly available case reports (CR) and evaluated them under two conditions: (1) on CR, representing the in-domain (ID) setting with the same document type as in training, and (2) on PN, representing the out-of-domain (OOD) setting with a different document type. This cross-document evaluation allows us to assess the models’ robustness to real-world variability in documentation styles, capturing the challenges introduced by the diverse writing practices found in in-hospital clinical notes.

Experimental results demonstrated that the fine-tuned LLM—specifically LLaMA-3.1 [14]—outperforms the MLM baseline (Bidirectional Encoder Representations from Transformers, BERT) [15], not only in the ID setting but also under OOD conditions. The LLM also exhibits a smaller performance gap between ID and OOD settings, indicating stronger robustness compared to the MLM. Further analysis reveals that LLMs are more resilient to stylistic diversity among clinicians, showing reduced performance fluctuation across different physicians. These findings underscore the potential of fine-tuned LLMs as more reliable tools for real-world clinical applications, particularly where robustness to diverse and previously unseen clinical notes is essential.

## Methods

### Overview

Our primary research question was to evaluate whether fine-tuned LLMs remain robust when applied to previously unseen clinical notes. To address this, we compared the performance gap, defined as the performance difference between ID setting and OOD settings between a fine-tuned LLM and a fine-tuned MLM, which serves as a state-of-the-art baseline for clinical NER.

**Figure 1.**
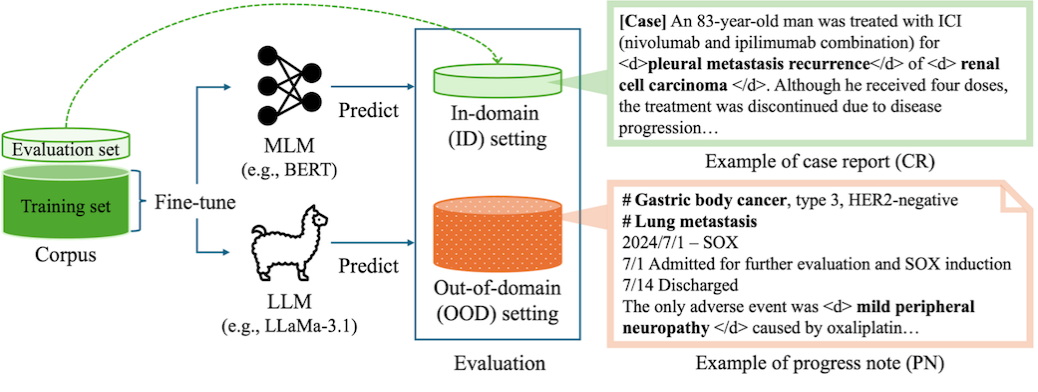
Overview of the evaluation pipeline. Models were fine-tuned on CR and evaluated on both CR, representing in-domain (ID) and PN, representing out-of-domain (OOD) test sets for disease name recognition in Japanese clinical notes. BERT: Bidirectional Encoder Representations from Transformers; HER2: human epidermal growth factor receptor 2; ICI: Immune checkpoint inhibitor; LLM: large language model; MLM: masked language model; SOX: SRY-related HMG-box.

The overview of the evaluation pipeline is shown in Figure 1. We evaluated model performance on the task of disease name recognition in Japanese clinical notes. Both LLMs and MLMs are first fine-tuned on a training set sampled from one document type (eg, case reports). Evaluation is then conducted on two distinct test sets: one ID set sampled from the same document type as the training data and one OOD set sampled from a different document type (eg, progress notes).

### Materials

To represent ID and OOD settings, we used two datasets: an existing publicly available corpus of case reports and a newly constructed corpus comprised of progress notes.

1. Case reports (CR): A publicly available dataset consisting of Japanese case reports comprising 1898 sentences across 148 documents annotated with clinical entities [16].
2. Progress notes (PN): A dedicated, newly constructed corpus of progress notes, comprising 1094 sentences across 100 documents annotated with disease entities.

For the construction of PN, we first curated 10 diverse board exam-style cases. To reflect real-world clinical documentation, these cases were then rewritten by ten physicians, each contributing ten unique documents. The physicians were instructed to adapt the cases to authentic in-hospital clinical note styles from their clinical practice, emphasizing realistic writing styles and varying levels of readability. In total, 1094 sentences (100 documents) across 10 physicians and 10 clinical cases were created. The created PN were then annotated for disease name entities by two experienced annotators, following the same annotation guidelines as CR [17]. We summarize the titles of the board exam-style cases and the top 3 most frequent disease entities per case in Table 1.

We assessed the annotation quality of PN by calculating interannotator agreement using two criteria: exact and partial span matching. Exact matching required both annotators to identify the same entity with identical span boundaries, while partial matching allowed for overlapping spans, acknowledging minor variations in boundary selection. Based on a comparison of annotations from 10 randomly sampled documents, the agreement between annotators was 0.70 (61/87) for exact matching and 0.82 (71/87) for partial matching, suggesting a high level of consistency and reliable annotation quality.

**Table 1.** Titles of the curated board exam-style cases and top 3 frequent disease entities.

Title and top 3 disease entities	Frequency (n)
“急性虫垂炎” (Acute appendicitis)	
“急性虫垂炎“ (Acute appendicitis)	11
“腹痛“ (Abdominal pain)	6
“穿孔や膿瘍“ (Perforation or abscess)	5
“進行胃がん“ (Advanced gastric cancer)	
“進行胃がん“ (Advanced gastric cancer)	8
“呼吸困難“ (Dyspnea)	7
“肝転移“ (Liver metastasis)	5
“肝膿瘍“ (Liver abscess)	
“肝膿瘍“ (Liver abscess)	15
“膿瘍“ (Abscess)	8
“倦怠感“ (Fatigue)	7
“月経過多に伴う貧血“ (Anemia due to menorrhagia)	
“子宮筋腫“ (Uterine fibroids)	20
“貧血“ (Anemia)	12
“月経過多“ (Menorrhagia)	8
“肺がん“ (Lung cancer)	
“肝転移“ (Liver metastasis)	8
“肺小細胞癌“ (Small cell lung cancer)	8
“低Na血症“ (Hyponatremia)	7
“IE (感染性心内膜炎）“ (Infective endocarditis)	
“くも膜下出“ (Subarachnoid hemorrhage)	8
SAH (Subarachnoid hemorrhage)	7
“疣贅“ (Vegetation - cardiac)	6
“誤嚥性肺炎“＋COPD^a^ (Aspiration Pneumonia with COPD)	
COPD	12
“誤嚥性肺炎“ (Aspiration pneumonia)	12
“浸潤影“ (Infiltrates)	6
“クモ膜下出血“ (Subarachnoid hemorrhage)	
SAH (Subarachnoid hemorrhage)	14
“頭痛“ (Headache)	9
“くも膜下出血“ (Subarachnoid hemorrhage)	5
“大腸がん“ (Colorectal cancer)	
“Isポリープ“ (Is polyp)	7
“大腸がん“ (Colorectal cancer)	7
Well-differentiated tubular adenocarcinoma in tubular adenoma	7
AMI (acute myocardial infarction)	
“胸痛“ (Chest pain)	15
AMI	8
“壁運動低下“ (Wall motion abnormality)	5

a COPD: chronic obstructive pulmonary disease

### Models and Baselines

We evaluate fine-tuned MLMs and LLMs on the task of disease name recognition in Japanese clinical notes, comparing their performance under ID and OOD settings. In addition, we evaluated LLMs using in-context learning (ICL) through zero-shot and few-shot prompting to assess the contribution of fine-tuning to improving LLM performance.

Fine-Tuning: We fine-tuned two models for the NER task: (1) “bert-base-japanese-v3” [18], based on BERT [15] as a strong MLM baseline, and (2) Swallow-Instruct-v0.2 [19], based on LLaMA-3.1 (version 8B; Meta) [14], which is a Japanese-instruction-tuned LLM. Both were subsequently fine-tuned on the ID training set. “bert-base-japanese-v3” was chosen as a strong and widely used baseline for Japanese-language tasks, providing a representative benchmark for traditional transformer–based MLMs that had been validated in clinical NLP [20]. In contrast, “Swallow-Instruct-v0.2” (version 0.2; Tokyo Institute of Technology) was selected to evaluate the potential of recent instruction–tuned LLMs, which are designed to better follow task-specific instructions and generalize across diverse inputs. Built on Llama 3.1 8B through continual pretraining, it was trained on a curated instruction corpus featuring multiturn dialogue and multilingual tasks with a particular focus on enhancing Japanese language capabilities.

For BERT, we adopted a standard sequence labeling approach, using the “CLS” token representations followed by a linear classification layer to predict BIO-tagged labels for disease entities. In contrast, for LLaMA-3.1, we followed a generation-based NER framework: the model was prompted with an instruction and clinical note, and it generated the same sentence with inline entity tags, enabling span-level extraction in a natural language generation format. To adapt LLaMA-3.1 to the NER task efficiently, we applied additional fine-tuning using Low-Rank Adaptation (LoRA) [21], allowing parameter-efficient fine-tuning without modifying the full set of model weights.

In-context learning (ICL): We evaluated LLaMA-3.1 and GPT-4o [22] under zero-shot and few-shot settings as baselines. An example of a prompt used for ICL is presented in Textbox 1. Recently, extensive efforts have been made to optimize prompt design in the field of NLP [23-26]. In this experiment, we provide the models with an annotation guideline that included entity definitions, task instructions, and illustrative examples, following prior work on medical NER [9]. For zero-shot learning, the model was provided with task instructions and annotation guidelines only, without any annotated examples. For few-shot learning, a single annotated clinical note was randomly selected from the training data and included in the prompt. Here, LLaMA-3.1 represents an open-source LLM with accessible model weights, allowing evaluation across both fine-tuning and in-context learning scenarios. In contrast, GPT-4o was evaluated only in zero-shot and few-shot settings due to its proprietary nature, serving as a reference for the in-context learning performance of an LLM with the highest model capacity in our study.

**Textbox 1. Prompt example for ICL.**

### Annotation Guideline

Definition of disease names

1. When the lesion or symptom has actually been observed in the patient
2. When it is suspected that the patient may have the lesion or symptom (eg, proposed as a differential diagnosis)
3. …

### Examples

Text: In November last year, pleural effusion appeared and increased, but decreased after starting furosemide.

Annotation: In November last year, <d pleural effusion </d>appeared and increased, but decreased after starting furosemide.

…

### Task Instruction

Based on the above explanation and examples, please annotate the following text

…

Text: The nodule shadow in the right upper lobe of the lung slightly increased.

### Comparison Settings

We used our clinical corpora in a cross-domain evaluation setup to assess both ID and OOD robustness:

ID: Samples from one document type were split 8:2 into training and evaluation sets. This setting reflects standard model development conditions, where training and test data share similar clinical notes.

OOD: The corpus from another document type was used in its entirety for evaluation. This reflects real-world deployment scenarios where models must process previously unseen clinical notes with varying writing styles and vocabularies.

In addition to the setting where CR are used for training and PN for evaluation, we also included the reverse scenario. This resulted in four distinct evaluation configurations: CR→CR, CR→PN, PN→PN, and PN→CR. The difference between ID and OOD performance (Δ) is calculated as Δ=ID−OOD. This represents the performance gap, which measures how well the model generalizes to unseen clinical notes, compared to the data it was trained on. A smaller difference indicates that the model is robust, even when faced with clinical notes with different writing styles or from different clinical cases. Hyperparameters used for fine-tuning are summarized in Table 2.

**Table 2.** Hyperparameters used for fine-tuning.

Parameter description	LLaMa-3.1	BERT^a^
Evaluation batch size per device	–^b^	8
Learning rate	2e-4	2e-5
LoRA^c^ dropout rate	0.05	–
LoRA rank (number of adaptation dims)	16	–
LoRA scaling factor	64	–
Maximum gradient norm (clipping)	0.3	–
Maximum sequence length	3000	512
Number of gradient accumulation steps	4	–
Number of training epochs	2	10
Optimizer used	AdamW	AdamW
Training batch size per device	4	8
Warmup ratio for learning rate	0.05	–
Weight decay for regularization	–	0.0
Number of warmup steps	–	0

a BERT: Bidirectional Encoder Representations from Transformers.

b Not applicable.

c LoRA: Low-Rank Adaptation.

### Ethical Considerations

This study did not involve experiments with human subjects, and no personally identifiable information was used at any stage. The clinical notes were physician-authored, based on board exam cases that are publicly available. Therefore, there are no ethical concerns related to patient privacy or informed consent in this research.

## Results

### Study Findings

Table 3 shows the findings of ID and OOD evaluation. All models were evaluated using the microaveraged *F*_1_-score, focusing on exact span matches of disease name entities. All results are averaged over three runs with different random seeds. For GPT-4o, we set the generation temperature to zero to ensure deterministic outputs and only ran the evaluation once due to annotation budget constraints.

**Table 3.** Evaluation results in micro *F*_1_-scores with standard deviations.

	CR^a^, mean (SD)			PN^b^, mean (SD)		
	→CR^a^ (ID)^c^	→PN^b^ (OOD)^d^	ΔCR^e^	→PN (ID)	→CR (OOD)	ΔPN^f^
LLaMA-3.1 (Zero-shot)	27.4 (0.3)	20.4 (0.1)	−7.0	15.5 (0.4)	27.1 (0.1)	11.6
LLaMA-3.1 (Few-shot)	32.6 (1.6)	30.5 (0.7)	−2.1	37.0 (4.8)	35.9 (0.5)	−11.0
GPT-4o (Zero-shot)	49.5 (0.0)	47.7 (0.0)	−1.8	42.0 (0.0)	50.8 (0.0)	8.8
GPT-4o (Few-shot)	53.4 (0.0)	49.9 (0.0)	−3.5	56.2 (0.0)	54.4 (0.0)	−1.8
BERT^g^(Fine-tuned)	73.7 (0.2)	59.8 (0.2)	−13.9	79.7 (1.8)	55.5 (1.7)	−24.2
LLaMA-3.1 (Fine-tuned)	78.4 (0.5)	69.8 (0.6)	−8.6	81.9 (0.7)	67.2 (0.5)	−14.7

a CR: case reports.

b PN: progress reports.

c ID: in-domain.

d OOD: out-of-domain.

e ΔCR: difference in csse reports.

f ΔPN: difference in progress notes.

g BERT: Bidirectional Encoder Representations from Transformers.

### Comparison Between Fine-Tuning and ICL

The fine-tuned LLaMA-3.1 consistently outperformed its zero-shot and few-shot counterparts, achieving the highest *F*_1_-scores across all evaluation settings. These results highlight the effectiveness of fine-tuning for clinical NER. In contrast, vanilla LLaMA-3.1 exhibited limited performance in zero-shot and few-shot scenarios. Notably, in the few-shot setting, the model showed a decline in performance when applied to OOD data.

GPT-4o demonstrated strong few-shot performance (ie, 53.4 on CR→CR and 56.2 on PN→PN) despite having no access to training data, highlighting the robustness of large-scale foundation models. However, these models still underperformed compared to the fine-tuned BERT, consistent with previous findings that task-specific fine-tuning often outperforms in-context learning in specialized domains like clinical NER [8,9]. These findings illustrate that while ICL can provide a competitive baseline with minimal data, fine-tuning remains essential.

### Comparison Between MLM and LLM

The fine-tuned LLaMA-3.1 outperformed the BERT baseline across all ID and OOD settings, demonstrating superior robustness to the previously unseen document type. Specifically, the fine-tuned LLaMA-3.1 exhibited a smaller performance gap (ΔCR=−8.6and ΔPN=−14.7) compared to BERT (ΔCR=−13.9 and ΔPN=−24.2), suggesting its greater stability for practical use in diverse clinical settings.

## Discussion

### Principal Findings

Experimental results demonstrate that the fine-tuned LLM exhibits strong robustness, maintaining relatively stable performance even when applied to previously unseen progress notes. To better understand the factors contributing to this robustness, we further decomposed it into two aspects: (1) robustness to stylistic variation, (ie, variations across physicians) and (2) robustness to variation across clinical cases. Our analysis reveals that the LLM was particularly robust to stylistic differences, while showing greater sensitivity to the differences in clinical cases.

We also conducted an error analysis to examine the qualitative improvements of the LLM over the MLM baseline performance. Our findings suggest that the LLM benefits from its generative approach, which allows it to mark entities inline within the sentence, as opposed to relying on a classification head over token representations, as in MLMs. This generative approach enables the LLM to more accurately extract entity spans, especially in stylistically diverse clinical notes.

### Impact of Physician and Clinical Case Variation

**Figure 2.**
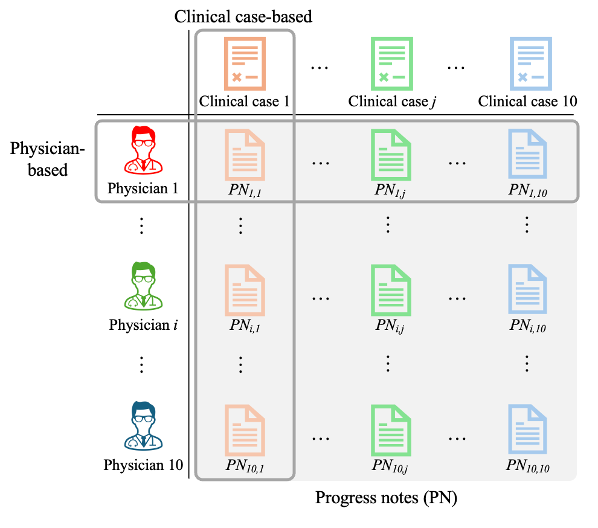
Overview of the evaluation with stratification. Performance of fine-tuned LLaMA-3.1 and BERT was stratified by physician and clinical case to assess robustness to writing style and clinical case variations.

To examine the robustness of the fine-tuned LLM to variations in writing styles and clinical cases, we stratified the performance of the fine-tuned LLaMA-3.1 and BERT in the CR→PN setting by physician and clinical case. The stratification process is summarized in Figure 2.

#### Physician-Based

Performance was evaluated for each physician, with ten clinical notes authored per individual. For instance, the performance of models is averaged across ${PN}_{i,1}$ to ${PN}_{i,10}$ for the i-th physician. The variation in these stratified results allows us to assess the model’s sensitivity to variations in individual writing styles.

#### Clinical Case-Based

Performance was also evaluated for each clinical case modeled after board exam-style scenarios (eg, acute appendicitis). The model’s performance was averaged over ten notes from different physicians, for example, ${PN}_{1,j}$ to ${PN}_{10,j}$ for the j-th clinical case. This stratification enables an analysis of the model’s ability to generalize to clinical-case-specific disease entities.

**Figure 3.**
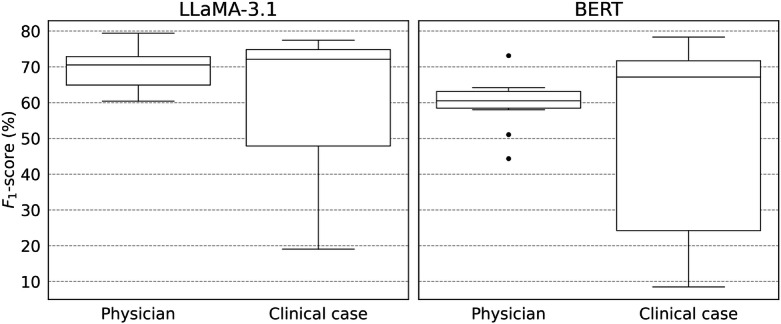
Distribution of *F*_1_-scores in CR→PN stratified by physician and clinical case. BERT: Bidirectional Encoder Representations from Transformers; CR: case report; PN: progress note.

Figure 3 presents the distribution of *F*_1_-scores stratified by physician and clinical case in the CR→PN setting for both the fine-tuned LLaMa-3.1 and BERT. The spread of each box and the range of whiskers reflect performance variability, while dots indicate outliers beyond 1.5-times the interquartile range. Narrower boxes and smaller ranges indicate higher consistency, while outliers and wider spreads highlight sensitivity to writing style or clinical case variation.

#### Physician Variance

The fine-tuned LLaMA-3.1 demonstrated consistently strong performance across different physicians, with relatively low variance in *F*_1_-scores. This is indicated in the narrow range between the maximum and minimum values in the box plot. In contrast, BERT’s performance varied more widely, with significant performance drops in some physicians’ notes, as indicated by extreme outliers and low minimum values. This suggests that the fine-tuned LLaMA-3.1 is more robust to stylistic differences, potentially due to its exposure to a broader and more diverse range of linguistic patterns during pretraining.

#### Clinical Case Variance

When stratified by clinical case, both models showed greater variability compared to writing style. This is evidenced by wider boxes and larger ranges between the maximum and minimum values in the box plots. These results highlight the increased difficulty of generalizing to domain-specific disease names. While the fine-tuned LLaMA-3.1 generally achieved higher average *F*_1_-score across clinical cases, it also experienced sharp drops in certain cases, indicating that it remains sensitive to clinical-case-specific variation. This underscores the persistent challenge in processing previously unseen clinical cases, even for large instruction-tuned models.

These findings underscore the relative strength of the fine-tuned LLaMA-3.1 in handling clinical stylistic variation. At the same time, they point to the need for further work in addressing performance gaps in different clinical cases.

### Error Analysis

To examine the qualitative improvements of the LLM over the MLM baselines, we conducted error analysis in the CR→PN setting. Based on the observation that the fine-tuned BERT has lower precision (51.8) compared to the fine-tuned LLaMA-3.1 (70.8), both models achieved similar recall scores (70.7 for BERT and 68.9 for LLaMA-3.1), we focused on false positive cases for the analysis.

Representative error examples are summarized in Table 4. From a manual inspection, we observed that BERT frequently misclassified nondisease clinical entities such as laboratory tests and biomarkers as disease mentions. For instance, in the sentence “Tumor markers also decreased gradually (徐々に腫瘍マーカーも低下し),” BERT incorrectly predicted two spans: “tumor markers (腫瘍マーカ”ー)” and “decrease (低下).” Both terms describe laboratory findings rather than disease entities. In another example, in the sentence “Therefore, with regard to platelets (このため血小板に対しては),” BERT erroneously extracted “platelets (血小板),” which refers to a blood cell type rather than a pathological condition. These examples illustrate that BERT often struggles to distinguish diagnostically relevant clinical phrases from true disease mentions, leading to false positive predictions.

In addition to entity type confusion, BERT also often struggled to capture the complete span of disease mentions, frequently producing boundary errors or partial matches that failed to align with the gold-standard annotations. For instance, in the sentence “Hb 8.1 g/dL and moderate nutritional disorder were observed (Hb8.1g/dlと中程度の栄養障害を認めた),” BERT incorrectly predicted only the prefix of a laboratory value, “Hb8,” entirely missing the actual disease mention “栄養障害 (nutritional disorder).” In another example, “The drainage volume was excessive (排液量が過多であった),” BERT extracted only the character “多 (excessive),” omitting the full phrase “排液量が過多 (excessive drainage volume).” These examples illustrate how slight shifts in input text can lead to misaligned token representations in MLM’s embedding space, resulting in fragmented or incomplete entity predictions.

In contrast, the LLM demonstrates greater flexibility in capturing complete spans of disease mentions. Unlike the MLM, which often struggles with partial matches, the LLM’s generative approach allows entities to be marked directly and seamlessly within the sentence. For example, in the sentence “Hb8.1g/dlと中程度の<d>栄養障害</d>を認めた” (translated as “Hb 8.1 g/dL and moderate <d>nutritional disorder</d>were observed”), the disease mention “栄養障害 (nutritional disorder)” is correctly and fully captured within the sentence using inline entity tags. This inline tagging strategy enables the model to stably extract entire disease names, even when clinical notes vary in writing style.

The LLM’s ability to overcome the errors observed in BERT predictions likely stems from its different learning paradigm: rather than relying solely on token-level classification based on fixed input embeddings, the LLM generates structured outputs conditioned on the full context of the input. This generative approach allows the LLM to better maintain entity span prediction coherence over varying document types and incorporate broader sentence-level semantics into prediction. Consequently, the LLM achieves more robust and accurate extraction performance compared to the MLM baseline, particularly in OOD settings.

**Table 4.** Examples of BERT prediction errors in the CR→PN setting. Each row shows a sentence (left), predicted entity spans (middle), and the correct gold annotations (right).

Example sentence (English/Japanese)	Prediction	Gold annotation
Tumor markers also decreased gradually/腫瘍マーカーも低下し	tumor markers/腫瘍マーカー, decrease/低下	None
Therefore, with regard to platelets/このため血小板に対しては	platelets/血小板	None
Hb 8.1g/dL and moderate nutritional disorder were observed/Hb8.1g/dlと中程度の栄養障害を認めた	Hb8	nutritional disorder/栄養障害
The drainage volume was excessive/排液量が過多であった	excessive/多	excessive drainage volume/排液量が過多

### Limitations

This study has several limitations. First, due to the annotation cost, our evaluation focused exclusively on disease name recognition. While disease entities are fundamental to clinical NLP tasks, real-world applications often require the extraction of a wider range of entities such as medications, procedures, and laboratory findings. Future research should expand the scope of entity types to provide a more comprehensive evaluation of model capabilities in diverse clinical information extraction tasks.

Second, we did not evaluate the computational efficiency or resource demands of the models. This is particularly relevant for LLMs, which often require substantial computational resources during both training and inference. Future studies should include a systematic comparison of computational cost, memory usage, and inference latency to guide more practical model deployment in clinical environments.

Lastly, we did not include comparisons with models pretrained on large-scale medical corpora, such as Bio-BERT [27] or its Japanese counterpart [28]. These models may have inherent advantages in understanding domain-specific terminology and context, and their inclusion could provide a clearer upper bound on MLM-based methods. Future work may incorporate medical-specific, pretrained models and a broader range of domain adaptation techniques in Japanese clinical settings.

### Conclusions

This study evaluated the performance of fine-tuned LLMs on disease name recognition in Japanese clinical notes, with a focus on both ID and OOD robustness. Our results demonstrate that fine-tuned LLMs, specifically the fine-tuned LLaMA-3.1, consistently outperforms the strong baselines across OOD settings, demonstrating superior robustness to previously unseen clinical notes.

Stratified analyses revealed that the LLM exhibits greater robustness to stylistic variation among physicians, as reflected in its lower performance variance across physicians. However, variations across clinical cases continue to pose significant challenges, with both LLM and the baseline model showing considerable fluctuations. Error analysis highlighted the LLM’s ability to consistently capture complete entity spans in stylistically diverse clinical notes. Its generative approach enables more context-aware span prediction, contributing to stable performance over the baseline model.

Overall, our findings underscore the potential of fine-tuned LLMs for clinical named entity recognition in low-resource languages such as Japanese, particularly in contexts with considerable variation in writing style. Nevertheless, challenges in cross-clinical case robustness remain. Future work should explore more targeted domain adaptation techniques and integration of external medical knowledge to further enhance robustness in real-world clinical NLP applications.

## Abbreviations

NLP: natural language processing
EHR: electronic health records
LLMs: large language models
ID: in-domain
OOD: out-of-domain
CR: case reports
PN: progress notes
MLM: masked language model
ICL: in-context learning
NER: named entity recognition
BIO: beginning inside outside
LoRA: low-rank adaptation
